# Cluttering in Children and Adolescents: Speech Motor Development, Neurocognitive Mechanisms, and Allied Health Implications

**DOI:** 10.3390/children13010097

**Published:** 2026-01-09

**Authors:** Weifeng Han, Lin Zhou, Juan Lu, Shane Pill

**Affiliations:** College of Education, Psychology and Social Work, Flinders University, Bedford Park, SA 5042, Australia

**Keywords:** cluttering, speech motor development, motor–cognitive integration, neurodevelopment, speech timing, developmental communication disorders

## Abstract

**Highlights:**

**What are the main findings?**

• Cluttering in childhood and adolescence reflects a developmental vulnerability in motor–cognitive integration, rather than a purely rate-based fluency disturbance.
• Symptoms emerge most clearly under high linguistic and cognitive load, demonstrating the central role of task demands and contextual variability in the expression of cluttering.

**What are the implications of the main findings?**

• Assessment should prioritise connected speech tasks that reveal timing instability, articulatory blurring and reduced monitoring, with attention to discourse and cognitive load.
• Intervention and future research should adopt developmentally informed, motor–cognitive frameworks capable of guiding more precise diagnosis, treatment and theoretical refinement.

**Abstract:**

**Background/Objectives:** Cluttering in childhood and adolescence is characterised by unstable speech timing, excessive coarticulation, irregular rate and reduced intelligibility, yet the developmental mechanisms underpinning these behaviours remain partially understood. This review synthesises empirical and conceptual evidence to examine cluttering through the lenses of speech motor development, neurocognitive mechanisms, task demands and allied-health practice. Four research questions guided the review, focusing on motor characteristics, developmental and neurocognitive mechanisms, task dependence and clinical implications. **Methods:** Following the PRISMA guidelines, a comprehensive search across seven databases identified studies examining cluttering in children and adolescents. Screening and full-text review were conducted in Covidence by two reviewers, with disagreements resolved by the first author. Twelve studies met the inclusion criteria. Data were extracted into a structured evidence table, and findings were synthesised. **Results:** Across studies, cluttering emerged as a developmental motor–cognitive integration disorder. Speech motor systems, linguistic formulation and executive control showed difficulty aligning under real-world communicative demands, leading to timing instability, articulatory blurring and reduced intelligibility. Symptoms were strongly influenced by task complexity, with spontaneous and extended discourse eliciting the most pronounced breakdowns. **Conclusions:** Cluttering reflects a developmental vulnerability in coordinating speech motor, linguistic and executive processes. Understanding cluttering in this way challenges narrow rate-based definitions and supports more nuanced approaches to assessment and intervention. Significant evidence gaps remain, particularly in longitudinal, mechanistic, multilingual and ecologically valid research. This developmental motor–cognitive framework strengthens the conceptual foundations of cluttering and clarifies its relevance to children’s motor development.

## 1. Introduction

Cluttering is a fluency disorder characterised by an abnormally rapid or irregular speech rate, excessive coarticulation, reduced intelligibility, atypical pausing, and deficits in self-monitoring [1]. Although traditionally discussed alongside stuttering, contemporary perspectives increasingly emphasise that cluttering reflects a distinct pattern of impaired speech motor control and cognitive-linguistic regulation, with symptoms often emerging during late childhood and adolescence [2]. The Limited Consensus Definition (LCD) [3] identifies three core features, i.e., perceived rapid or irregular speech rate, atypical fluency, and reduced intelligibility resulting from over-coarticulation or collapsing syllables, all supported by additional characteristics such as deficits in pragmatic, linguistic, and executive functioning.

Understanding cluttering from a developmental perspective is of importance for two reasons. First, speech is a highly specialised motor skill that undergoes maturation throughout childhood and adolescence. Motor development frameworks [4] emphasise the progressive stabilisation of timing, sequencing, coordination, and automatization, i.e., domains directly implicated in cluttering. Dynamic systems models (e.g., [5]), describe speech as the coordinated integration of multiple subsystems (respiratory, phonatory, articulatory, prosodic), each sensitive to cognitive load and task demands. When system demands exceed developmental capacity, instability, variability, and breakdowns occur. This motor-development lens highlights the interplay between perceptual, cognitive, linguistic, and motor processes across maturation. Cluttering, when viewed as an emerging speech motor coordination disorder with neurodevelopmental roots [6], aligns naturally with this focus.

Assessment represents a central challenge in the study of cluttering, particularly in children and adolescents [7]. Unlike more narrowly defined fluency disorders, cluttering is typically identified through patterns observed in connected speech, including variable speech rate, reduced intelligibility, excessive coarticulation, and limited self-monitoring, rather than through discrete, task-independent markers [8,9]. As a result, assessment practices often rely on extended discourse tasks and clinical judgement, and may vary substantially across settings. These challenges highlight the importance of understanding how speech motor development, linguistic and cognitive load, and task demands interact to shape the manifestation of cluttering and underscore the need for a clearer developmental framework to inform assessment decisions.

However, emerging evidence indicates that cluttering rarely exists in isolation. Its behavioural presentation often reflects the combined influence of speech motor control, linguistic planning, executive functioning, and broader neurodevelopmental profiles. For example, Bangert, Scott [10] identified cluttering-like features in children with fragile X syndrome, and suggested that atypical timing and working memory may contribute to fluency breakdowns. Fidan and Sarıyer [7] reported that children with cluttering show weaknesses in attention, working memory, and motor planning abilities, particularly in those with co-occurring attention-deficit/hyperactivity disorder (ADHD). Scott [11], Scott [12] provided developmental insights into rapid speech, over-coarticulation, and contextual variability across tasks, while Kelkar [1] and Duchan and Felsenfeld [13] emphasised the need for integrated motor, linguistic, and pragmatic assessment frameworks. Collectively, current evidence underscores the importance of examining cluttering as a multidimensional, developmentally sensitive, and context-dependent phenomenon.

Despite these advances, major gaps remain. Most studies addressing speech motor control in cluttering have been conducted with adults (e.g., [14]), therefore limiting their applicability to childhood development. Paediatric research is scarce and heterogeneous, with few studies exploring underlying motor or neurocognitive mechanisms directly. Few studies used kinematic, neuroimaging, or physiological measures to examine motor coordination or timing processes. Instead, existing paediatric literature relies largely on behavioural description, or indirect indices of cognitive load and motor planning (e.g., [15]). This gap limits our understanding of how speech motor control, linguistic formulation, and executive processes interact during critical periods of motor and cognitive growth.

Given these limitations, there is a need for a comprehensive synthesis and analysis of what is currently known about the speech motor, neurocognitive, and neurodevelopmental mechanisms underpinning cluttering in children and adolescents, and that situates these mechanisms within contemporary motor development frameworks. Such a review is essential for informing allied health practice, refining assessment, and guiding future intervention research grounded in motor-learning principles. Moreover, an integrative perspective is essential for improving early identification, particularly for children whose cluttering co-occurs with ADHD, fragile X syndrome, developmental language disorder, or other neurodevelopmental conditions (see, for example, [7,10,16,17]). A developmental motor–cognitive model may also inform school-based practice and educational participation that will ensure young people with cluttering receive support that aligns with their communication needs and developmental profile.

This review is intended as a systematic synthesis with a structured narrative and developmental focus, aiming to clarify patterns, constraints, and gaps in an emerging literature rather than to establish empirically validated causal models. It was guided by four interrelated research questions that together form the conceptual framework of the manuscript. Specifically, we asked: how speech motor characteristics are described in children and adolescents who clutter (RQ1); what developmental and neurocognitive mechanisms are proposed to underpin these characteristics (RQ2); how task demands and contextual factors shape the expression of cluttering (RQ3); and what implications these findings have for allied-health assessment and intervention (RQ4). Each section of the review is explicitly aligned with one or more of these questions, enabling a structured synthesis of a heterogeneous evidence base and a clear argumentative progression from description to interpretation and application.

## 2. Methods

This review employed a systematic search strategy combined with a structured narrative synthesis to examine cluttering in children and adolescents. While systematic procedures were used to identify, screen, and select relevant studies, a meta-analytic approach was not appropriate due to substantial heterogeneity in study designs, participant characteristics, outcome measures, and theoretical orientations. Instead, findings were synthesised narratively using a domain-based framework aligned to the four research questions, allowing integration of empirical, developmental, and clinically informative evidence while preserving conceptual coherence. Our procedures followed PRISMA guidelines [18,19], and the screening process was facilitated using Covidence [20] to ensure consistency and reproducibility.

### 2.1. Search Strategy

We conducted a comprehensive search of four major databases commonly used in communication sciences and allied health research: Scopus, PsycINFO, PubMed, and CINAHL. Our search terms combined cluttering-related descriptors (such as “cluttering”, “fluency disorder”, “rapid rate”, “irregular rate”, “over-coarticulation”), motor and neurocognitive terminology (including “motor planning”, “speech motor”, “timing”, “coarticulation”, “executive function”, “working memory”, “attention”, etc.), and developmental descriptors (e.g., “child”, “children”, “adolescent”, “school-aged”, “developmental”). Boolean operators and truncation ensured high sensitivity across databases. We supplemented database searching with manual screening of reference lists of key cluttering publications and forward citation tracking of foundational cluttering papers to ensure full coverage of relevant empirical studies. Table 1 and Table 2 outline the PICO framework, key concepts and terms that facilitated the search.

### 2.2. Inclusion and Exclusion Criteria

To ensure that the final corpus addressed RQ1–RQ4 directly and reflected contemporary definitions of cluttering, we applied the inclusion and exclusion criteria summarised in Table 3. These criteria were developed prior to screening and applied consistently throughout. These criteria ensured that each included study contributed directly to at least one of the four research questions.

Importantly, studies published from the year 2000 onwards were included to ensure alignment with contemporary diagnostic frameworks, assessment practices, and developmental models of speech motor and neurocognitive functioning. Earlier literature, while foundational in defining cluttering as a clinical construct, often predates greater consensus regarding diagnostic criteria and task-based assessment approaches, limiting its direct comparability with more recent developmental research.

Studies were also excluded if the term “cluttering” was used without accompanying descriptions consistent with established behavioural characteristics of cluttering, such as irregular or rapid speech rate, reduced intelligibility in connected speech, excessive coarticulation, or limited self-monitoring. This criterion was applied to ensure conceptual consistency across included studies. Decisions regarding exclusion on this basis were made through independent screening by two reviewers, with discrepancies discussed and resolved through consensus, and final determinations made by the first author.

Given the limited number of empirical paediatric studies directly examining cluttering, the review included multiple types of scholarly sources, including theoretical, conceptual, and clinically grounded papers, provided they addressed at least one of the research questions. These sources were not treated as equivalent to empirical studies; rather, they were used to inform the interpretation of developmental mechanisms, contextualise empirical findings, and articulate implications for assessment and intervention. This approach reflects established practice in integrative developmental reviews where empirical evidence is sparse or methodologically heterogeneous.

### 2.3. Study Screening and Selection

**Figure 1:**
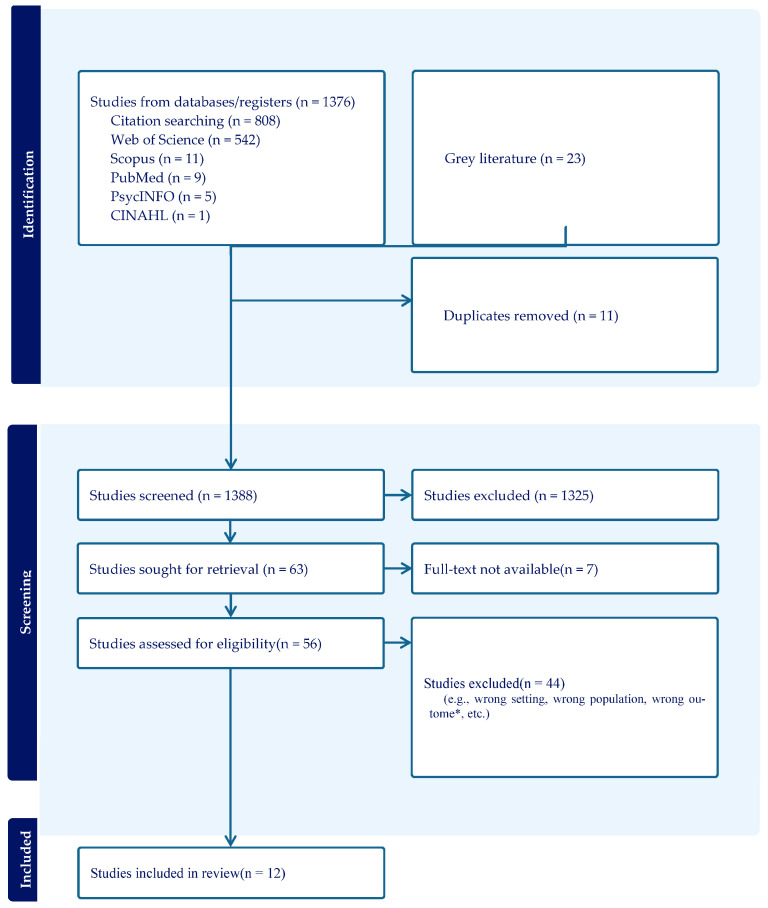
PRISMA chart. * Note. Research of wrong outcomes denotes those that did not report motor, neurocognitive, or task/context outcomes relevant to RQ1–RQ4, including Adult-only sample; that did not examine cluttering (term used without cluttering-consistent behaviours); or that did not report outcomes relevant to motor/neurocognitive/task-demand aims (RQ1–RQ4).

All search results were imported into Covidence for automated deduplication. The screening process followed the PRISMA framework [18,19]. Title and abstract screening was conducted independently by the second and third authors, who applied the eligibility criteria to each record. Full-text screening followed the same procedure. When disagreements occurred, the first author resolved conflicts through discussion and final decision-making. Twelve studies were included in the final synthesis and analysis, which provided the evidence foundation for addressing RQ1, RQ2, RQ3 and RQ4. The PRISMA flow diagram (Figure 1) provides a detailed account of the number of records identified, screened, excluded and retained.

### 2.4. Data Extraction and Synthesis

Once the final corpus was established, the first author extracted all data into a structured template that captured study design, participant characteristics, definitions of cluttering, speech motor, neurocognitive or contextual features addressed, and primary findings. Each study was also mapped to the research questions it informed. Our synthesis proceeded by grouping extracted findings according to the four research questions: RQ1 (speech motor development), RQ2 (neurocognitive and neurodevelopmental mechanisms), RQ3 (task and contextual influences) and RQ4 (allied-health assessment and intervention implications).

Given the heterogeneity of the cluttering literature, synthesis was guided by explicit differentiation criteria rather than statistical aggregation. Studies were interpreted according to their primary analytic focus and developmental relevance, including empirical investigations of paediatric speech motor and cognitive characteristics, developmentally informative mechanistic studies, and clinically grounded conceptual or assessment-oriented work. This distinction informed a structured narrative synthesis aligned to the research questions, enabling identification of converging patterns, areas of divergence, and levels of evidential support across domains while avoiding inappropriate homogenisation of clinically and methodologically diverse sources.

Notably, the included literature reflects an emerging evidence base with substantial methodological diversity. To support transparent synthesis, we interpreted findings using an evidence-tier approach. Empirical paediatric studies were treated as direct evidence for observable speech motor and task-related characteristics. Mechanistic or developmentally informative work was used to propose functional interpretations, without being treated as causal evidence for paediatric mechanisms. Conceptual and clinical literature were used to clarify definitions, assessment considerations, and plausible pathways linking speech motor and cognitive demands, with inferences explicitly identified as interpretive rather than empirically established. This approach allowed integration across domains while keeping claims aligned with the strength and type of evidence available.

To support transparency and provide a clear overview of the evidence base, Table 4 summarises the twelve included studies. For each study, we summarise study design, participant characteristics, focal domains and their contribution to RQ1–RQ4. The table provides a point of reference for the subsequent synthesis and highlights the methodological diversity of the existing literature on cluttering in children and adolescents.

## 3. Developmental and Theoretical Foundations of Cluttering

Cluttering during childhood and adolescence unfolds within a developmental period marked by ongoing refinement of speech motor control, linguistic organisation and executive functioning. These systems mature at different rates, and their interaction forms the foundation for understanding the speech characteristics and the contextual influences addressed in RQ1 and RQ3. By situating cluttering within a developmental motor framework, we can interpret the varied behaviours observed in children who clutter and understand why symptoms fluctuate across tasks and contexts.

Speech production is among the most temporally complex motor behaviours acquired in childhood [26]. Throughout the school years, children continue to refine articulatory timing, coarticulation and prosodic control as their speech motor patterns stabilise. Developmental research (e.g., [27,28]), shows that children’s articulatory gestures are more variable, their segmental timing is less consistent, and their prosodic patterns are more easily disrupted than those of adults. These patterns reflect a motor system still consolidating automatization [29].

This developmental context is essential for understanding cluttering. Paediatric research shows that children who clutter exhibit timing variability, imprecise articulation and instability in rhythm, particularly during spontaneous or complex speech [11,12]. Such findings align with developmental speech literature (e.g., [30,31]), that when linguistic formulation demands increase, a less stable motor system becomes more vulnerable to irregular timing and articulatory “blurring”. Cluttering becomes especially salient during the school years, when communicative and cognitive expectations increase, and children are required to produce longer, more complex verbal output.

Although rapid or irregular speech rate is often emphasised clinically, cluttering is widely understood as a multidimensional phenomenon involving the interaction of motoric, linguistic and pragmatic processes [1]. Historical analyses outline cluttering as encompassing imprecise articulation, disorganised verbal output and reduced self-monitoring [13], while more recent descriptions highlight excessive coarticulation, unstable prosody and difficulty coordinating extended discourse [11]. Consensus work underscores that no single feature defines cluttering; rather, individuals present with different constellations of symptoms that shift across contexts [2,6,21,24,25].

This multidimensionality is reflected across the included studies. Children who clutter not only speak rapidly or irregularly but often show reduced monitoring, challenges with discourse organisation and difficulty maintaining intelligibility across tasks [11,12]. Evidence from neurocognitive comparisons indicates that weaknesses in executive functioning or working memory can compound motor difficulties [7], therefore reinforcing the need to understand cluttering as the outcome of interacting systems rather than a purely motoric disorder.

Speech timing and sequencing are also central to the motor profile of cluttering. Empirical work shows that individuals who push speech beyond their capacity limits experience timing breakdowns and decreased intelligibility [22]. In children who clutter, timing instability is especially evident during connected speech production, where the motor system must coordinate rapid sequences while managing linguistic formulation demands. Rapid bursts, uneven rhythm and variable segment durations have been documented in school-aged children [12], particularly during tasks requiring spontaneous or extended speech.

Theoretical accounts also support these observations. Models of speech motor control describe cluttering as involving vulnerabilities in systems responsible for temporal regulation and articulatory coordination [24] and potential inefficiencies in neural circuits associated with rhythm and sequencing [21]. Together, these models and empirical findings suggest that cluttering reflects a developmental weakness in achieving stable, well-regulated timing, especially when cognitive or linguistic load increases.

In general, fluency disorders arise from the interplay between speech motor control, linguistic organisation and executive functioning [32]. Weaknesses in any of these domains can, therefore, amplify demands on the others. For example, rapid linguistic formulation may exceed the motor system’s capacity to execute articulatory sequences with precision, leading to excessive coarticulation or syllable collapse [33]. Similarly, unstable motor execution may increase the cognitive load required to monitor speech, reducing efficiency in discourse organisation [34].

The included studies provide evidence of these cross-domain interactions. Children with ADHD who clutter show greater difficulty with executive functioning and working memory than their non-cluttering peers [7], evidencing how neurocognitive load can intensify motor variability. Studies also show that cluttering behaviours often increase during narrative or expository tasks that require sustained planning and monitoring [11,12]. These findings are consistent with developmental research and demonstrate that fluent speech in complex tasks depends on well-coordinated cognitive, linguistic and motor systems.

When cluttering is viewed through a developmental motor lens, a coherent interpretation emerges, i.e., cluttering reflects an imbalance in the synchronisation of speech motor, linguistic and cognitive subsystems. Dynamic systems theories emphasise that motor output becomes less stable under increased cognitive load or rapid transitions [5], precisely the conditions under which cluttering features are most prominent. Structured tasks that reduce linguistic demands often minimise cluttering behaviours, while spontaneous discourse and complex communicative activities reveal the system’s vulnerabilities. Understanding cluttering as a dynamic coordination disorder, therefore, clarifies why symptoms fluctuate across tasks and contexts and prepares the ground for examining the neurocognitive mechanisms that underpin these patterns.

Taken together, findings across these studies converge on the presence of instability in speech timing and articulatory coordination, particularly during connected speech, despite variability in study design and measurement approaches. While empirical paediatric studies provide direct evidence of these features, clinically oriented and mechanistic accounts offer complementary insights into how such motor instability may emerge under increased linguistic and cognitive load. Differences across studies appear to reflect task demands and analytic focus rather than contradictory findings, supporting a coherent interpretation of speech motor disruption as a central feature of cluttering.

## 4. Speech Motor Characteristics of Cluttering in Childhood and Adolescence

The empirical studies included in this review offer converging evidence on the speech motor characteristics of cluttering in children and adolescents and directly inform RQ1 and RQ3. Although the evidence base is modest and varied in scope, clear patterns emerge across studies: children who clutter demonstrate instability in speech timing and rhythm, variability in rate regulation, reduced articulatory precision and pronounced task-dependent fluctuations. These findings complement the conceptual foundations established in Section 3 and illustrate how developmental vulnerabilities across motor, linguistic and cognitive systems manifest in observable speech behaviours.

Timing instability is one of the most consistent findings across the included studies. Research has demonstrated that when speakers exceed their speech motor capacity, timing becomes irregular and intelligibility declines [22]. Although the study involved clients across the life span, its capacity-demand framework is directly applicable to developmental populations. In school-aged children, research found that cluttering symptoms were marked by variable syllable durations, abrupt shifts in tempo and irregular rhythmic patterns [12]. These timing disruptions were especially evident during spontaneous and linguistically complex tasks and were less prominent in structured activities. Descriptive synthesis [11] reinforces this pattern, noting that inconsistent rhythm and uneven timing frequently co-occur with reduced intelligibility in children who clutter. Together, these findings show that timing instability is not a superficial feature of cluttering but a core indicator of underlying vulnerability in temporal regulation systems. These instabilities also form a bridge to RQ2, where timing-related mechanisms are examined more directly from a neurocognitive and neurodevelopmental perspective.

Irregular or excessively rapid speech rate is another key behavioural marker of cluttering, and the included studies emphasise difficulties regulating rate rather than deliberate speaking fast. Children who clutter often accelerate unintentionally, which suggests limited capacity to modulate output in real time. For example, children who clutter were found to produce rapid bursts of speech during narrative and expository tasks, even when explicitly cued to reduce rate [12]. These uncontrolled accelerations were accompanied by reductions in intelligibility and increased articulatory imprecision. Speech, therefore, deteriorates when speakers operate beyond their individual capacity thresholds [22]. Rate regulation difficulties in cluttering thus reflect disruptions in the dynamic balance between linguistic formulation and motor execution. These findings support the developmental motor framework outlined previously and illustrate how regulation demands increase as communicative complexity grows.

Further, reduced intelligibility in cluttering primarily arises from excessive coarticulation and imprecise or incomplete articulatory gestures. Paediatric data (e.g., [11,12]), show that children who clutter produced blurred articulatory transitions and collapsed syllables, particularly during longer, less structured speech tasks. These articulatory patterns were less evident in word-level responses, therefore indicating that cluttering becomes more salient as linguistic and cognitive demands increase. Similarly, the motor speech framework [24] describes cluttering as involving excessive anticipatory coarticulation and reduced segmental precision. Although not focused exclusively on children, this framework is consistent with empirical observations in paediatric samples and aligns with developmental research showing that coarticulation patterns continue maturing through adolescence. When combined with irregular timing and inadequate monitoring, excessive coarticulation contributes significantly to intelligibility breakdowns in children who clutter.

One of the clearest findings across the included studies is that cluttering symptoms vary substantially across speaking tasks. For example, cluttering behaviours tend to intensify during spontaneous and cognitively demanding contexts, whereas more structured tasks yield fewer or milder symptoms [12]. It is, therefore, important to sample connected speech across a range of communicative contexts to capture the full breadth of cluttering behaviours in school-aged children. That means task-dependent variability is not diagnostic noise; it is a defining characteristic of cluttering. This aligns with developmental models of motor coordination, which show that stability decreases as cognitive or linguistic load increases. The evidence, therefore, indicates that cluttering cannot be fully understood without considering the interaction between motor capacity and communicative context.

Several included studies also highlight the relevance of cognitive and neurodevelopmental factors in amplifying motor instability. For example, children with ADHD who clutter are found to exhibit greater weaknesses in executive functioning and working memory than peers with ADHD who did not clutter [7]. These findings suggest that cluttering in some children may arise from combined motor and executive vulnerabilities, which can influence rate regulation, timing consistency and self-monitoring.

Further insights come from research on neurodevelopmental conditions that present with cluttering-like characteristics. Rapid speech, timing irregularities and segmental imprecision were also documented in individuals with fragile X syndrome [10]. The evidence supports the possibility that cluttering shares characteristics with broader timing and sequencing difficulties observed in neurodevelopmental disorders. These findings lay the groundwork for understanding the neurobiological mechanisms.

## 5. Neurocognitive and Neurodevelopmental Mechanisms Underpinning Cluttering

Cluttering emerges during a developmental period characterised not only by the refinement of speech motor control but also by substantial growth in executive functioning, working memory, linguistic integration and neural efficiency. RQ2 seeks to clarify how neurocognitive and neurodevelopmental factors interact with motor control to shape cluttering behaviours in children and adolescents. While empirical paediatric studies provide direct evidence of observable speech motor and task-related features, conceptual and clinically oriented work offers complementary perspectives on how these features may arise developmentally and how they manifest in practice. Although the included studies provide limited direct neurological data, they collectively point to a set of convergent mechanisms involving timing, sequencing, executive control, processing speed and self-regulation. These mechanisms help explain why cluttering varies across tasks and why symptoms often intensify during spontaneous or cognitively demanding communication.

Timing and sequencing processes underpin fluent speech and rely on the coordinated activity of cortical and subcortical neural pathways. Theoretical models highlight the involvement of cortico-basal ganglia-cerebellar circuits in rate control and temporal precision [21], and disruptions in these systems have long been proposed as contributors to cluttering. Although the included studies do not provide direct neurophysiological evidence, their behavioural findings strongly suggest underlying timing vulnerabilities. Observations of inconsistent rhythm, variable segment durations and rapid bursts, for example, indicate reduced temporal stability in children who clutter [12]. The evidence suggests that cluttering-like features may emerge when neural timing mechanisms are compromised. Collectively, these findings align with the developmental motor perspective outlined earlier, i.e., children with cluttering may have a narrower margin of temporal stability, causing motor coordination to break down when cognitive or linguistic load increases.

Fluent speech also requires continuous coordination of linguistic formulation, monitoring and real-time motor sequencing. Executive functions, and working memory in particular, play a key role in managing these processes. Evidence from the included studies demonstrates that children who clutter may present with executive function vulnerabilities that intensify speech motor instability. For example, research found that children with ADHD who cluttered showed weaker working memory and executive control than children with ADHD who did not clutter [7]. Because working memory supports planning, sequencing and linguistic coherence, reduced capacity can lead to rapid, poorly monitored speech and disorganised utterances. Furthermore, executive control assists in regulating rate, inhibiting premature articulatory gestures and adjusting output based on listener feedback. When these capacities are compromised, rate regulation becomes difficult, monitoring is inconsistent, and cluttering emerges more readily, particularly with complex communication needs [35]. This neurocognitive interpretation fits well with observations that individuals who clutter may outpace their planning during speech [36]. Rather than attributing cluttering solely to a fast rate, as a result, our findings support that cognitive-linguistic processes and motor planning become misaligned when executive resources are insufficient to manage the load.

Evidence from children’s speech supports that in cluttering, linguistic planning outpaces the motor system’s ability to execute articulatory sequences with stability. For example, documented cases where children appeared to generate linguistic content rapidly, leading to compressed syllables, excessive coarticulation and reduced intelligibility [12] are consistent with a planning-execution mismatch, i.e., when linguistic content is generated more quickly than motor commands can be precisely implemented, speech becomes blurred and irregular. Such a mismatch may be further compounded by children’s developing neural systems. For example, neural efficiency increases throughout childhood and adolescence [37], and processing speed in both linguistic and motor domains improves with age [38]. Therefore, when these systems do not develop synchronously, timing conflicts can arise [39]. The evidence reviewed suggests that cluttering manifests when linguistic formulation is rapid but motor control is insufficiently stabilised, which results in an unstable output pattern.

Another critical component of fluent communication is self-monitoring, the ability to attend to one’s speech in real time and adjust output accordingly. Multiple studies and clinical descriptions note that individuals who clutter often show reduced self-monitoring or limited awareness of speech breakdowns [6,11]. This reduced monitoring may reflect limitations in attention allocation or in the integration of auditory and proprioceptive feedback. Self-monitoring demands increase substantially during extended discourse, where children must maintain coherence, adjust rate and attend to listener needs simultaneously. When monitoring systems are taxed, children may fail to detect rapid rate increases or articulatory imprecision until the breakdown is already perceptible to the listener. Executive vulnerabilities may, therefore, limit not only planning but also retrospective awareness, creating a cycle in which cluttering behaviours persist without timely self-correction.

Although cluttering can occur independently, several neurodevelopmental conditions present with cluttering-like speech characteristics. For example, rapid rate, irregular timing and imprecise articulation are found in individuals with conditions associated with atypical neural connectivity and developmental timing differences [10]. Such findings suggest that cluttering behaviours may arise when underlying neural systems involved in sequencing and rhythm are affected. Studies examining cluttering in the context of ADHD provide similar insights. Similarly, executive function weaknesses were also found to contribute to increased cluttering behaviours [7]. These results suggest that cluttering may, in some children, reflect broader neurodevelopmental vulnerabilities rather than isolated speech motor disruption. These observations, however, do not imply that cluttering is secondary to other developmental conditions; rather, they highlight shared mechanisms, such as timing instability, working memory load, and reduced monitoring, that may manifest across different neurodevelopmental profiles.

In interpreting these findings, it is important to distinguish between observed behavioural characteristics and the mechanisms proposed to account for them. While disruptions in speech timing and articulatory coordination are directly observed, for example, associations with linguistic formulation, attentional control, and executive functioning are inferred from task effects and developmental patterns rather than demonstrated as causal relationships. These associations are therefore best understood as functionally linked dimensions within a broader developmental system rather than as isolated or hierarchically ordered deficits.

The mechanisms described above, therefore, converge to support an integrated account of cluttering in childhood and adolescence. Cluttering appears to arise from the interaction of (a) timing and sequencing vulnerabilities, which reflect inefficiencies in neural circuits responsible for temporal regulation; (b) executive function and working memory limitations, which impair planning, attention shifting and monitoring; (c) processing speed mismatches, which cause linguistic formulation to outpace motor stability; and (d) reduced self-monitoring, which limit the ability to regulate rate and correct breakdowns. These interacting vulnerabilities provide a coherent explanation for the speech motor patterns and the contextual sensitivity observed across studies. They also align with developmental models emphasising that speech fluency depends on synchronised contributions from cognitive, linguistic and motor systems. A pattern we found from the synthesis is that when these systems develop asynchronously or operate under increased load, the risk of cluttering behaviours increases.

## 6. Assessment and Intervention Implications for Allied Health Practice

The empirical and conceptual evidence in earlier sections has clear implications for how cluttering should be assessed and managed within allied-health practice. RQ4 focuses on identifying elements of assessment and intervention that are supported by the existing evidence base and are appropriate for children and adolescents, whose speech motor, cognitive and linguistic systems are still developing. Although the twelve included studies vary in design and purpose, they converge on a set of clinically meaningful insights that can guide practice.

Our findings have implications for assessment. Across multiple included studies, cluttering symptoms were most evident during spontaneous or extended speech [11,12]. Rapid rate, timing instability, excessive coarticulation and reduced intelligibility often diminished in structured tasks but returned during longer monologic or narrative speech. For assessment, this underscores the need to obtain samples across a range of contexts, including conversation, narrative and expository tasks. Reliance on single-word or heavily scaffolded tasks is likely to under-identify cluttering behaviours.

Rate variability and unintentional acceleration are prominent in children who clutter. Findings that speech deteriorates when speakers exceed their capacity limits [22] suggest that assessment should examine not only habitual rate but also rate flexibility and behaviours under increased load. This may involve having the child shift between speaking rates, sustain a slower rate during complex tasks or demonstrate rate control while simultaneously organising linguistic content.

Children who clutter also often present with reduced intelligibility caused by blurred articulatory transitions and excessive coarticulation [12,24]. Assessment should therefore include careful analysis of articulatory precision, segmental clarity and syllable integrity within naturalistic speech. Because intelligibility breakdowns frequently emerge only under specific conditions, clinicians must attend to task-based variability rather than relying solely on averaged scores or structured elicitation.

Evidence (e.g., [7]), shows that children who clutter may have weaknesses in working memory and executive control. Although SLPs do not formally diagnose executive function disorders, they can observe behaviours such as difficulty sustaining attention, rapid shifts between ideas, limited awareness of communication breakdowns and challenges organising multi-clause discourse. These behaviours provide essential contextual information when forming a differential diagnosis and determining whether cluttering emerges primarily from motor, cognitive-linguistic or combined vulnerabilities.

Cluttering often co-occurs with other communication or developmental conditions, including stuttering, ADHD and, in some cases, broader neurodevelopmental differences [10,11]. Assessment procedures should therefore incorporate differential diagnosis protocols that consider shared symptoms (such as rapid rate or disfluencies) while distinguishing cluttering from related disorders based on timing instability, coarticulatory excess, monitoring behaviours and task-dependent variability. Interviews with families and teachers can provide insights into when cluttering behaviours are most noticeable and how they affect functional communication in school and social settings.

The findings also share implications for intervention. Current evidence shows that because rate instability is a core feature of childhood cluttering, rate control remains a central therapeutic focus. However, the evidence also suggests that therapy should not aim merely to “slow speech,” but rather to support children in developing regulation of rate across tasks and contexts. For example, research notes that children often accelerate unintentionally even when aware of the need to slow down, which indicates limited self-regulation rather than a preference for fast speech [12]. Intervention should therefore integrate strategies such as pausing cues, pacing boards, rhythmic supports, metronomic guidance and visual counters, used flexibly to promote internalised tempo regulation.

Excessive coarticulation and imprecise articulation contribute significantly to reduced intelligibility in cluttering. Motor-based strategies such as over-articulation practice, syllable stretching, targeted contrast drills and explicit focus on articulatory gestures may help strengthen clarity. For example, research highlights the value of combining rate control with articulatory cueing, which allows children to allocate sufficient time for complete gestural execution [24,25].

Many children who clutter have reduced awareness of breakdowns. Intervention should include activities that enhance the child’s ability to monitor their speech in real time, evaluate intelligibility and adjust output. Approaches may involve video or audio playback, supported self-rating scales, and guided reflection activities. The ultimate importance is to help children recognise when their speech accelerates or becomes disorganised and develop strategies for re-establishing control [11].

Given the evidence linking cluttering to working memory and executive function challenges [7], therapy may be strengthened by incorporating supports that reduce cognitive load or increase predictability. These may include organising frameworks for narrative tasks, visual planning for longer utterances, chunking strategies and scaffolds that promote sustained attention. While not executive-function training per se, these supports may improve fluency by aligning linguistic planning with motor execution.

The clinical implications outlined here are grounded primarily in converging behavioural evidence and established developmental principles, rather than in direct experimental manipulation of underlying mechanisms, and should be interpreted accordingly. Furthermore, because cluttering behaviours shift across tasks, intervention should be delivered across a variety of communicative contexts. Structured tasks may help children develop foundational skills, but therapy must eventually extend into spontaneous discourse, functional classroom activities and real-life conversational contexts. This approach helps ensure that improvements generalise to settings where cluttering is most pronounced.

When cluttering co-occurs with other communication or developmental conditions, intervention may require adaptation. For example, when ADHD is present, therapy may integrate brief, highly engaging activities, predictable routines and external cues to support sustained attention. For young people with broader neurodevelopmental differences, intervention may need to address sensory, attentional or pragmatic challenges that influence speech motor stability. Cross-disciplinary research (e.g., [10]), suggests that cluttering-like behaviours in some populations may reflect underlying neurobiological timing vulnerabilities, underscoring the importance of interdisciplinary collaboration.

Finally, viewing cluttering through a developmental lens highlights the importance of synchronising assessment and intervention with a child’s evolving motor, cognitive and linguistic capacities. The combined evidence suggests that cluttering is best supported through approaches that capture variability across contexts, focus on rate regulation and articulatory stability, build self-monitoring, scaffold executive functioning demands, and accommodate co-occurring conditions when present.

## 7. General Discussion

This review set out to examine cluttering in children and adolescents from four interrelated angles: speech motor development (RQ1), neurocognitive and neurodevelopmental mechanisms (RQ2), task and contextual influences (RQ3), and implications for allied-health assessment and intervention (RQ4). Across the twelve included studies, a coherent picture emerges. Cluttering in childhood and adolescence is best understood as a developmental disorder of motor–cognitive integration, in which speech motor systems, executive processes and linguistic planning fail to synchronise efficiently under real-world communicative demands. Importantly, the synthesis presented here distinguishes between types of evidence and their respective contributions, integrating empirical paediatric findings with developmentally informative mechanistic and clinical literature to build a coherent account of cluttering without conflating distinct levels of analysis. In what follows, we integrate findings across Section 3, Section 4, Section 5 and Section 6 to articulate this developmental account, highlight the central role of task demands, and consider implications for theory, classification and practice. Where explanatory mechanisms are discussed, these are framed as integrative, developmentally informed interpretations that organise existing evidence and generate testable hypotheses, rather than as definitive accounts of causality.

Rather than treating heterogeneity as noise, therefore, we operationalised variability in cluttering along analytically relevant dimensions that are consistently implicated across studies: developmental stage, diagnostic practice, task demands (structured speech vs. spontaneous discourse), and cognitive–linguistic load. This typology supports the interpretation of why cluttering features vary across contexts and explains why connected speech tasks are most informative for identifying timing instability, coarticulatory blurring, and reduced self-monitoring. Importantly, this framework does not assume discrete subtypes on current evidence; instead, it provides a structured way to interpret variation while highlighting priorities for future mechanistic and longitudinal research.

### 7.1. Cluttering as a Developmental Motor–Cognitive Disorder

The evidence reviewed across Section 3, Section 4 and Section 5 indicates that cluttering cannot be reduced to a single domain of impairment. Rather than a purely motor speech disorder or a purely cognitive-linguistic difficulty, cluttering in childhood appears where developing systems for motor timing, linguistic formulation, and executive regulation intersect and occasionally misalign. Children who clutter are navigating the same maturational processes as their peers: refinement of speech motor patterns, growth in linguistic complexity and increasing demands on planning and self-regulation. What distinguishes them is the fragility of the coordination among these systems when communicative demands are high.

From a developmental perspective, this coordination problem is unsurprising. Speech requires fine-grained temporal control while simultaneously accommodating lexical retrieval, syntactic assembly, discourse organisation and monitoring of listener needs. The studies reviewed suggest that in children who clutter, temporal and articulatory systems are more vulnerable to disruption when executive resources are stretched, working memory is taxed or linguistic formulation becomes rapid and dense. The characteristic “blurring” of segments, irregular bursts of speech and fluctuating intelligibility are thus not isolated symptoms but the surface expression of a deeper integration difficulty.

This account also helps clarify how cluttering is similar to, and distinct from, other fluency and motor speech disorders. Like stuttering, cluttering appears to involve vulnerabilities in timing and sequencing [2]; like some dysarthrias or developmental coordination disorders, it reflects challenges in achieving stable motor patterns [29]. Unlike these conditions, however, cluttering is defined by its close coupling to discourse-level formulation and monitoring. The speech motor system does not fail in isolation; rather, it fails when asked to keep pace with the child’s linguistic and cognitive tempo. This motor–cognitive integration view, therefore, offers a unifying framework that honours the multidimensional descriptions of cluttering without fragmenting it into unrelated symptom clusters.

Importantly, conceptualising cluttering as a developmental integration disorder also aligns with work in motor development [40], which emphasises that complex motor behaviours emerge from the interaction of multiple subsystems rather than from a single locus of control. In this sense, cluttering offers a valuable case study for motor development research as it shows how high-level cognitive and linguistic processes can destabilise a maturing motor system, and how motor constraints can, in turn, shape the expression of language and discourse.

### 7.2. The Central Role of Task Demands and Environmental Load

A second major insight from this review is the centrality of task demands and environmental load in shaping how cluttering is expressed. Section 4 documented that cluttering behaviours are most evident during spontaneous, extended and linguistically rich speech, and attenuated in highly structured or constrained tasks. Section 5 linked this task sensitivity to the limited capacity of developing executive systems to manage planning, sequencing and monitoring under pressure. Taken together, these findings suggest that cluttering is not simply a property of the individual. It is rather equally a property of the interaction between the child and the communicative environment. The implications outlined below are therefore presented as practice-relevant inferences grounded in converging behavioural evidence and developmental theory, while acknowledging that direct mechanistic validation in paediatric samples remains a priority for future research.

This interactional view has important conceptual implications. If cluttering emerges most reliably when children must generate ideas, structure discourse, monitor their listener and regulate their rate simultaneously, then variability across tasks is not a nuisance variable but a defining feature of the condition. It follows that “inconsistency” in cluttering across contexts should not be interpreted as diagnostic unreliability, but as evidence that the underlying vulnerability is highly sensitive to load. This perspective reframes longstanding clinical frustrations, such as children who “do not clutter” during a short structured assessment but clutter extensively in the classroom, as predictable outcomes of the capacity-demand balance.

The role of environmental load extends beyond task structure. Classroom expectations, assessment practices and interactional norms can either mask or reveal cluttering. For example, tightly scaffolded question-answer formats may fail to elicit cluttering behaviours, whereas open-ended show-and-tell, oral presentations and narrative tasks are much more likely to expose the limits of motor–cognitive coordination. Similarly, noisy environments, rapid turn-taking, or time pressure may all increase the risk of cluttering behaviours in children whose systems are already working near capacity.

Recognising this dependence on task and context has methodological implications for research as well. Studies relying solely on reading tasks or brief picture descriptions will underestimate the presence and severity of cluttering, especially in younger speakers. Research designs that incorporate ecologically valid tasks, such as curriculum-based oral activities or semi-structured peer interactions, are more likely to capture the phenomena that concern children, families and teachers. In this way, the cluttering literature highlights a broader lesson for motor development research, i.e., understanding a developing system requires studying it under the kinds of loads it routinely experiences, not just under idealised clinical conditions.

### 7.3. Implications for Theory, Classification and Clinical Practice

A third contribution of this review lies in its implications for how cluttering is conceptualised and classified, and how these conceptual choices shape clinical practice. Traditional definitions anchored in rapid or irregular rates are increasingly at odds with the multi-component picture emerging from empirical and clinical work. Rate instability remains central, but on its own it is insufficient to differentiate cluttering from other fluency disturbances or from typical developmental variability in fast-talking children.

The integrated account developed here suggests that cluttering is better characterised by a constellation of features, including vulnerability in temporal regulation of speech, a tendency for linguistic formulation to outpace stable motor execution, reduced effectiveness of monitoring under high load and marked contextual variability, etc. Such a profile does not map neatly onto existing diagnostic categories, which often separate “motor” and “language” disorders as if they were independent. A developmental motor–cognitive integration framework provides a more accurate description and encourages classification systems to recognise cluttering as a disorder that sits at the interface of motor speech and developmental neurocognition.

Clinically, this shift has several consequences. For assessment, it supports the use of multi-context, discourse-level tasks, explicit attention to timing and coarticulation and targeted observation of monitoring and executive behaviours. It also justifies integrating information from teachers and families, who observe the child in precisely the high-load settings where cluttering is most evident. For intervention, it encourages programmes that go beyond rate control drills to encompass self-monitoring, discourse planning and management of cognitive load.

There are also implications for team-based practice. Because cluttering often co-occurs with broader developmental differences, such as ADHD or neurogenetic conditions, SLPs may need to work closely with psychologists, paediatricians, educators and, where appropriate, neurologists. A shared understanding of cluttering as a motor–cognitive integration difficulty allows these professionals to situate cluttering within the child’s overall developmental profile rather than treating it as a discrete symptom to be “fixed” in isolation. This, in turn, supports more coherent planning of supports across home, school and clinic.

Finally, this integrated perspective has consequences for how cluttering is communicated to children and families. Framing cluttering as a difficulty in coordinating fast-moving systems, rather than as a flaw in character or effort, can reduce stigma and support more collaborative goal-setting. It also provides a rationale for why certain situations feel particularly challenging and why therapy may focus on building skills for managing complex communicative demands rather than simply “slowing down.”. It should also be pointed out that the integrative account advanced in this review does not assume direct causal relationships between linguistic, cognitive, attentional, and neurodevelopmental dimensions. Instead, cluttering is conceptualised as emerging from the dynamic interaction of these processes during speech production, particularly under conditions of increased communicative load. Empirical studies provide evidence for observable speech motor instability and task sensitivity, while developmental and neurocognitive accounts offer plausible explanatory frameworks for these patterns. Explicitly distinguishing observed phenomena from proposed mechanisms allows a coherent synthesis without overstating the strength of causal inference.

### 7.4. Gaps and Future Directions

The integrated account presented here reveals several gaps and opportunities in the current evidence base. It was necessary for this review to exclude studies that used the term “cluttering” without describing cluttering-consistent behaviours, which highlights a broader issue within the literature. Inconsistent or imprecise use of terminology complicates comparison across studies and contributes to uncertainty in prevalence estimates, assessment practices, and research synthesis. This lack of terminological consistency represents an important gap in the field and underscores the need for clearer operational definitions and reporting standards in future research on cluttering, particularly in paediatric populations.

Another striking limitation is the scarcity of longitudinal work. Most studies offer snapshots of cluttering at a single point in time, leaving open questions about how motor–cognitive integration evolves across development, why some children appear to “grow out of” cluttering and how intervention influences trajectories. Longitudinal studies following children from early school years into adolescence would allow researchers to examine whether cluttering reflects a transient delay in integration, a persistent vulnerability or multiple developmental pathways. Nevertheless, it is worth pointing out that the small number of empirical paediatric studies underscores the limited state of mechanistic research on cluttering in childhood, rather than a weakness of the present review. Accordingly, this synthesis integrates empirical findings with developmentally informative theoretical and clinical literature to construct a coherent account of cluttering as a motor–cognitive integration difficulty. Empirical evidence anchors the description of observable phenomena, while non-empirical sources contribute to theory-building and clinical interpretation, allowing conclusions to be calibrated to the available evidence.

Methodologically, there is a need for studies that combine detailed behavioural observations with more fine-grained measures of timing, sequencing and neural function. Acoustic and kinematic techniques could elucidate how temporal patterns in cluttering differ from those in other speech motor disorders. Neuroimaging or electrophysiological methods, used judiciously, therefore, could begin to map the neural correlates of motor–cognitive coordination difficulties. At present, mechanistic accounts remain largely inferential; more direct evidence would allow the field to refine or revise current models.

There is also a notable gap in cross-linguistic and multilingual research. Given that cluttering involves interactions between motor control and linguistic structure, languages with different phonological, prosodic or morphosyntactic profiles may shape its expression in distinct ways [41]. Studies in non-English languages, particularly those that are typologically different from English [42], and in children acquiring more than one language [43], would not only broaden the empirical base but also test the generalisability of the motor–cognitive integration model proposed here. Such work would be particularly valuable in contexts where multilingualism is the norm and where cluttering may be under-recognised or misattributed to second-language learning difficulties.

Variability in diagnostic practices, sample sizes, and task paradigms across studies, therefore, necessarily constrains the generalisability of individual findings, which underscores the importance of interpreting convergence across domains rather than relying on any single study or methodological approach.

A further underdeveloped area concerns real-world participation and quality of life. For example, there is limited research on how cluttering affects children’s academic engagement, peer relationships and self-concept, or how these outcomes change with intervention. Integrating participation-level outcomes into research designs would align cluttering research more closely with contemporary frameworks in speech-language pathology and with the priorities of the clients, their families and care teams [44], as well as the integration of modern technologies [45].

Finally, future work would benefit from closer integration with the broader motor development literature. The challenges of coordinating speech under high cognitive load are mirrored in other motor domains, such as complex limb movements [46] or bimanual tasks [47]. Collaborative research across speech science, motor control and developmental psychology could yield richer models of how motor–cognitive integration develops and why it sometimes falters. Future reviews may also build on this work by examining the historical evolution of cluttering concepts in earlier literature, particularly to trace how definitional and diagnostic shifts have shaped contemporary research and practice. In this way, cluttering research can both draw from and contribute to a wider understanding of children’s motor development.

## 8. Conclusions

In this review, we synthesised developmental, motor, cognitive, and clinical evidence to examine cluttering in children and adolescents. Across the twelve included studies, a consistent picture emerged, i.e., cluttering reflects a developmental vulnerability in the coordination of speech motor timing, linguistic formulation, and executive control. These findings address all four research questions by clarifying the speech motor characteristics of cluttering (RQ1), identifying the developmental and neurocognitive mechanisms that underpin them (RQ2), demonstrating the central role of task and contextual demands in symptom expression (RQ3), and outlining the implications for allied-health assessment and intervention (RQ4).

By framing cluttering as a motor–cognitive integration disorder, we strengthened its alignment with contemporary theories of children’s motor development. Cluttering arises most clearly when linguistic, cognitive, and motor systems must operate simultaneously under load, revealing the limits of an integration process that is still maturing. This perspective challenges rate-based definitions and supports a more coherent model in which temporal instability, articulatory blurring, reduced monitoring, and contextual variability are understood as interdependent manifestations of a single developmental mechanism.

While the available empirical literature remains limited, the synthesis presented here integrates behavioural findings with developmentally informative theoretical and clinical work to advance a coherent framework for understanding cluttering in childhood and adolescence. In the meantime, the evidence base remains limited, particularly in relation to paediatric samples, longitudinal trajectories, mechanistic studies, and multilingual contexts. Addressing these gaps will be essential for refining theoretical models, informing classification systems, and improving clinical practice. Even with these constraints, the developmental motor–cognitive account advanced here provides a foundation for future research and a clearer rationale for assessment and intervention in both clinical and educational settings.

Cluttering is often under-recognised, misunderstood, or misclassified. By articulating cluttering as a developmental motor–cognitive integration difficulty, this review advances conceptual clarity in the field and provides a foundation for more precise assessment, intervention, and future research in children’s motor development.

## Figures and Tables

**Table 1:** PICO framework.

Element	Description
Population	Children and adolescents (0–18 years) with cluttering
Intervention	Speech motor control, motor learning, neuromotor development, or neurological correlates
Comparison	Typically developing peers, or other fluency/motor disorders (e.g., stuttering, CAS, dysarthria)
Outcomes	Speech fluency, intelligibility, prosody, motor timing/coordination, neurological or genetic findings, intervention outcomes

**Table 2:** Concepts and search terms. Asterisks (*) are used as a truncation or wildcard symbol to broaden the search by retrieving various forms and spellings of a word from a single root term, which is a standard/default practice in systematic searches of literature.

Concept	Key Terms
Cluttering/Fluency Disorder	“clutter*”, “fluency disorder”, “disfluency”, “rate disorder”, “tachylalia”, “rapid speech”, “stutter*”
Motor control/Motor speech	“motor control”, “motor timing”, “motor coordination”, “motor learning”, “speech motor control”, “speech movement”, “articulatory control”, “neuromotor”
Neurodevelopment	“neurodevelopment”, “developmental disorder”, “neural correlates”, “neurological”, “cerebellar”, “basal ganglia”, “executive function”, “genetic”
Comparative disorders	“stutter*”, “childhood apraxia of speech”, “CAS”, “dysarthria”, “speech sound disorder”
Allied health/intervention	“speech pathology*”, “speech therap*”, “occupational therap*”, “physiotherapy*”, “interven*”, “motor learning”, “rehab*”, “communica*”
Population	“child*”, “adolescen*”, “youth”, “p?ediatric”, “school-age*”

**Table 3:** Inclusion and exclusion criteria.

Inclusion Criteria	Exclusion Criteria
Research examining cluttering in children or adolescents (≤18 years), or mixed-age samples with extractable paediatric data	Studies involving adults only
Cluttering is defined using behavioural indicators (e.g., rapid or irregular rate, excessive coarticulation, reduced intelligibility, atypical fluency)	Studies describing fluency or motor speech disorders unrelated to cluttering (e.g., isolated stuttering, dysarthria, or CAS)
Evidence relevant to speech motor development, neurocognition, neurodevelopmental mechanisms, contextual task effects or allied-health clinical practice	Studies using the term cluttering without describing cluttering-consistent behaviours
Peer-reviewed studies published after 1 January 2000	Non-peer-reviewed studies; or studies published before 2000
Published in English	Non-English studies
No geographical restrictions	

**Table 4:** Summary of included studies.

Study	Type/Design	Age Range	Focus	Motor/Neurodevelopmental Emphasis	Allied-Health Relevance	Relevance to RQs
Alm [21]	Theoretical paper	Lifespan (developmentally relevant)	Neurobiological mechanisms	Speech motor timing; cortico-basal ganglia circuits	Rate/timing assessment considerations	RQ1, RQ2
Bakker, Myers [22]	Empirical (cross-sectional)	Adults with developmental relevance	Motor performance under rate demands	Capacity-based motor control	Rate control; monitoring	RQ1, RQ3, RQ4
Bangert, Scott [10]	Empirical (cross-sectional phenotype study)	Young adults with FXS	Neurodevelopmental phenotype	FXS-related timing, cognition, and disfluency	Complex-needs assessment	RQ2, RQ4
Drayna [17]	Evidence synthesis	Lifespan	Genetic and heritable factors	Neural circuitry; familial aggregation	Family history; referrals	RQ2
Duchan [6]	Conceptual paper	Lifespan	Multilayered clinical construct	Motor-language-pragmatics interaction	Contextualised assessment	RQ1, RQ3, RQ4
Duchan and Felsenfeld [13]	Theoretical paper	Lifespan	Historical evolution of cluttering	Motor + cognitive shifts over time	Diagnostic implications	RQ1, RQ4
Fidan and Sarıyer [7]	Empirical (comparative)	Children/adolescents with ADHD	EF, WM, reading, cluttering	ADHD-linked executive and motor-planning difficulties	Integrated cognitive-motor assessment	RQ2, RQ4
Kelkar, Sanghi [23]	Instrument validation	SLPs (Speech Language Pathologists)/consumer samples	Impact of cluttering	Participation-level impacts	Outcome measurement in practice	RQ4
Scott [12]	Empirical (child speech study)	School-aged children	Contextual variability in cluttering	Motor timing + discourse load	Task-based assessment	RQ1, RQ3
Scott [11]	Clinical/educational discussion	School-aged children	Symptomatology and management	Motor, cognitive, and linguistic features	Assessment, differential diagnosis	RQ1, RQ3, RQ4
Ward [24]	Theoretical paper	Lifespan	Motor speech mechanisms	Timing, articulation, prosody	Motor-focused intervention	RQ1, RQ3, RQ4
Ward [25]	Clinical practice paper	Lifespan	Motor-focused treatment	Rate control, over-articulation	Clinically actionable strategies	RQ3, RQ4

## Data Availability

No new data were created or analyzed in this study. Data sharing is not applicable to this article.

## Abbreviations

The following abbreviations are used in this manuscript:

ADHD	Attention-Deficit/Hyperactivity Disorder
LCD	Limited Consensus Definition
SLP	Speech-Language Pathologist
